# Respiratory Monitoring by Ultrafast Humidity Sensors with Nanomaterials: A Review

**DOI:** 10.3390/s22031251

**Published:** 2022-02-07

**Authors:** Shinya Kano, Nutpaphat Jarulertwathana, Syazwani Mohd-Noor, Jerome K. Hyun, Ryota Asahara, Harutaka Mekaru

**Affiliations:** 1 Human Augmentation Research Center, National Institute of Advanced Industrial Science and Technology (AIST), Kashiwa 277-0882, Japan; h-mekaru@aist.go.jp; 2 Sensing System Research Center, National Institute of Advanced Industrial Science and Technology (AIST), Tsukuba 305-8563, Japan; 3 Department of Chemistry and Nanoscience, Ewha Womans University, Seoul 03760, Korea; nj181qng02@ewhain.net (N.J.); syazwani@ewhain.net (S.M.-N.); kadam.hyun@ewha.ac.kr (J.K.H.); 4 Human Informatics and Interaction Research Institute, National Institute of Advanced Industrial Science and Technology (AIST), Tsukuba 305-8566, Japan; ryo-asahara@aist.go.jp

**Keywords:** humidity sensor, fast response, nanomaterial, respiratory monitoring, physiological monitoring

## Abstract

Respiratory monitoring is a fundamental method to understand the physiological and psychological relationships between respiration and the human body. In this review, we overview recent developments on ultrafast humidity sensors with functional nanomaterials for monitoring human respiration. Key advances in design and materials have resulted in humidity sensors with response and recovery times reaching 8 ms. In addition, these sensors are particularly beneficial for respiratory monitoring by being portable and noninvasive. We systematically classify the reported sensors according to four types of output signals: impedance, light, frequency, and voltage. Design strategies for preparing ultrafast humidity sensors using nanomaterials are discussed with regard to physical parameters such as the nanomaterial film thickness, porosity, and hydrophilicity. We also summarize other applications that require ultrafast humidity sensors for physiological studies. This review provides key guidelines and directions for preparing and applying such sensors in practical applications.

## 1. Introduction

The respiration system of the human body is controlled by the respiratory center in the brain, which regulates the amount of oxygen (O_2_) and carbon dioxide (CO_2_) in arterial blood [1]. Respiratory behavior reflects human health and physical/mental activity [2,3,4]. Physical load on the body raises respiratory rates and air volume because oxygen consumption and carbon dioxide production are increased by exercising muscles [5]. Negative emotions with fear and anxiety increase respiratory rates [6,7,8,9]. To understand the physiological and psychological relationships between respiration and the human body, various methods to observe the respiratory response have been proposed [10,11,12,13]. There are mainly two categories of devices: those that take measurements with/without contacting the body. Most conventional devices contact the chest, abdomen, or head, and read electrical, acoustic, ultrasonic, or mechanical signals. Recently, fully noncontact monitoring of human respiration was proposed by using radio-frequency waves [14,15].

Humidity is also a valuable observable for contact-based health monitoring. Humidity sensors with ultrafast response and recovery times have been recently proposed in the field of materials science [16,17]. Functional nanomaterials play an important role in speeding up the response and recovery rates of the sensors. As the human breath has a high humidity content, ultrafast humidity sensors have the potential to follow respiration patterns in real time and provide vital health information. Various demonstrations of respiratory monitoring in subjects using humidity have been reported [17]. In this review, we overview recent developments on ultrafast humidity sensors using functional nanomaterials for respiratory monitoring. Four working principles of humidity sensors are introduced: impedance, light, frequency, and voltage-based mechanisms. Selected demonstrations that highlight their potential or progress in respiratory monitoring are described. We compare the response/recovery times of recently reported humidity sensors and discuss their applicability in respiratory monitoring. Finally, other examples that observe water-related physiological phenomena using ultrafast humidity sensors are given.

## 2. Respiration Measured by Humidity Sensor

### 2.1. Indicators of Respiration

**Figure 1:**
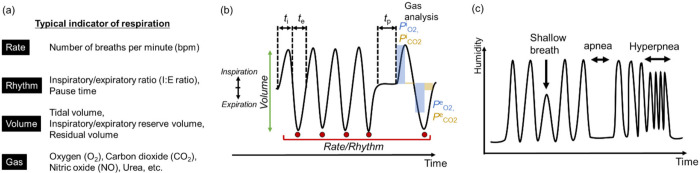
(**a**) Indicators of respiratory system. (**b**) General features in typical respiratory signals from sensors. *t*_i_: time of inspiration, *t*_e_: time of expiration, *t*_p_: pause time, *P*^i^_O2,CO2_: partial pressure of O_2_/CO_2_ in inspiratory air (ambient air), *P*^e^_O2,CO2_: partial pressure of O_2_/CO_2_ in respiratory air. (**c**) Example of correlations between humidity and irregular breathing patterns.

There are four fundamental indicators that characterize respiration: respiratory rate, rhythm, volume, and gas components, as shown in Figure 1a. In this subsection, we summarize what information can be obtained from each indicator. Respiratory rate is clinically important to check the vitality of patients [18]. The number of breaths per minute (bpm) is used for describing the respiratory rate. One of the simplest methods is for medical staff to count the number of respirations of a patient by eye for 15 s and then convert the number to a respiratory rate [18]. The respiratory rate is dependent on age. A normal respiratory rate for a healthy adult ranges from 10 to 18 bpm [19,20,21]. In general, children have faster respiratory rates than adults (30 bpm for the age of 0–1 year old) [18,22]. An abnormal respiratory rate is induced by many pulmonary diseases such as chronic obstructive pulmonary disease and asthma [18,23]. Cretikos et al. proposed that an adult with a respiratory rate of 20 bpm was not unwell and over 24 bpm had some critical illness [3].

It is possible to evaluate the breathing rhythm by calculating the ratio of inspiratory (*t*_i_) to expiratory times (*t*_e_), known as the inspiratory/expiratory ratio (I:E ratio) [7]. Adjusting the I:E ratio is required in mechanical ventilation systems for maintaining spontaneous breathing [20]. Some respiratory symptoms show typical patterns of respiration, for example, Cheyne–Stokes respiration and Biot’s respiration [21,24]. Yuan et al. summarized well-known abnormal respiratory patterns, which are indicative of injured respiratory centers in the brain, use of narcotic medications, metabolic derangements, and weak respiratory systems [1]. The pause time (*t*_p_) between inspiration and expiration is another indicator that describes a disorder in respiratory patterns [7]. If the pause time is too long, the subject may be suffering from apnea. Mental effort with concentration can suppress the variability in respiratory patterns as distraction leads to irregular respiration [7]. For evaluation of rhythm, it is critical to measure the time of respiratory phases precisely. 

Analysis of volume and gas components in breath air is another tool for understanding the health of subjects. The volume of breath air can be evaluated by several factors: tidal volume, inspiratory/expiratory reserve volume, and residual volume. The depth of breathing corresponds to the volume. These factors are measured by a spirometer and used to evaluate the functionality of the lung. The function of breathing is to exchange gas components with the blood through pulmonary alveoli: taking O_2_ into and excreting CO_2_ from the body [11]. In general, partial pressures of O_2_ and CO_2_ in ambient air (i.e., inspiratory air) are *P*^i^_O2_ = 159 mmHg and *P*^i^_CO2_ = 0.3 mmHg, and those in expiratory air are *P*^e^_O2_ = 120 mmHg and *P*^e^_CO2_ = 27 mmHg, respectively [25]. The concentration of O_2_ and CO_2_ can be used for evaluating metabolisms in the body. Specific gas components are used to find diseases in clinical breath analysis. The nitric oxide (NO) concentration on a ppb scale increases due to airway inflammation from asthma [26]. The urea breath test is useful for the detection of helicobacter pylori infection in the stomach, which can be a risk factor for gastric cancer [27]. In analyzing the volume and gas of exhaled air, it is also critical to make quantitative and temporal measurements. These features can be obtained by analyzing the signal from spirometry, plethysmography, and capnography, as noted in the schematic plot of Figure 1b.

Expiratory air has nearly saturated moisture (47 mmHg at 37 °C). By using humidity sensors in front of the human mouth/nose, it is possible to monitor the change in humidity between exhaled and ambient air. For example, Figure 1c schematically shows correlations between humidity and irregular breathing patterns. Apnea and hyperpnea can be found from the patterns of peaks. When the response speed of the humidity sensor is insufficient, the amplitude of peaks tends to decrease under hyperpnea. For smaller humidity changes from shallow breathing, the corresponding peak height decreases.

### 2.2. Concept to Monitor Respiration with Humidity-Sensitive Nanomaterial

**Figure 2:**
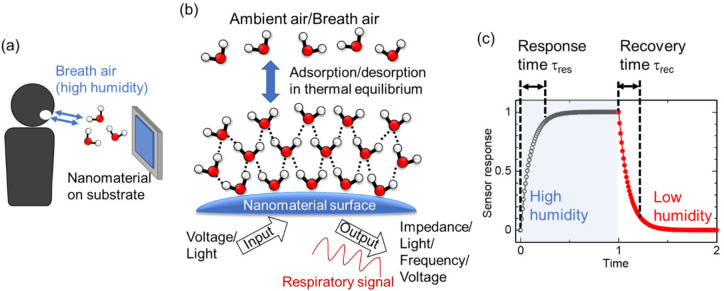
(**a**) Schematic illustration of respiratory monitoring by humidity-sensitive nanomaterials. Nanomaterials on the substrate are directly exposed to breath air with water molecules. (**b**) Microscopic structure of adsorbed water molecules on the nanomaterial surface. (**c**) Definition of response and recovery time as *t*_90_ in the ideal response of humidity sensors.

Using nanomaterials for gas sensors is advantageous for obtaining high sensitivity to adsorbates because of their large surface-to-volume ratio and beneficial structure–property relationships (e.g., enhanced signals from nanostructures due to surface plasmon resonances) [28]. Humidity-sensitive nanomaterials have been applied to respiratory monitoring, as schematically shown in Figure 2a. A film of humidity-sensitive nanomaterial is deposited on a substrate by physical/chemical methods. The easiest method for preparation is to coat the substrate with a colloidal solution of the nanomaterial as size-regulated nanomaterials are usually produced by chemical synthesis in solvent. When the film is exposed to breath air from the nose and mouth, water molecules in the air adsorb onto the nanomaterial surface. Breath air carries much more moisture than usual ambient air because the inspiratory air is humidified in the nose and the lung. The adsorption and desorption of the molecules are in thermal equilibrium at the nanomaterial surface, as shown in Figure 2b. We can sense the adsorbed water molecules by observing a change in one or more physical properties of the nanomaterial. The inputs of the sensor are usually voltage and light. The typical outputs of the sensor signals are impedance, light, frequency, and voltage, as explained later. If the response and recovery are rapid enough to track the exchange between ambient and breath air, we can obtain respiratory signals as a continuous wave in the output. 

**Figure 3:**
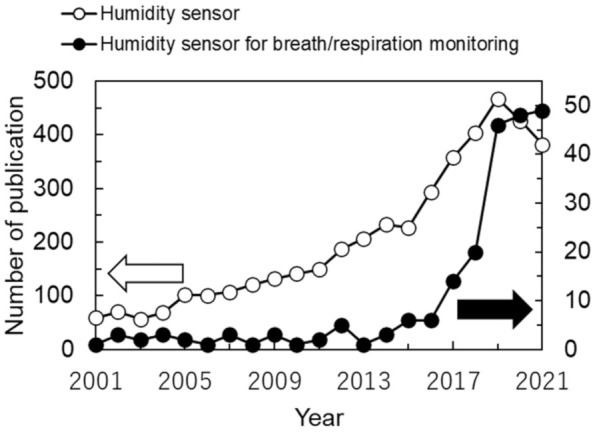
Recent trends of the number of publications reporting humidity sensors for respiratory monitoring (filled circles). The number of publications relating to humidity sensors is also shown as open circles. The data were surveyed on 7 January 2022 in Web of Science using the filter: (“humidity sensor”) AND (“breath” OR “respiration”).

The response and recovery times provide a standard to evaluate the quickness of the sensor response. Figure 2c graphically defines the response and recovery time. In the figure, an ideal sensor response is shown when the sensor is exposed to high humidity (open circles) then low humidity (closed circles), successively. A typical indicator is *t*_90_, which is the time required for the signal to reach 90% of the total change. A similar indicator is *t*_63_, where the signal must reach 63% of the total change (corresponding to a time constant (1-1/*e*)*: e* is the Napier’s constant). It is obvious that *t*_90_ is longer than *t*_63_, and, therefore, using *t*_90_ corresponds to a stricter evaluation of the response. When we apply the humidity sensors to respiratory monitoring, it is preferable that the response time τ_res_ and recovery time τ_rec_ are shorter than 60/2*f*_R_, where *f*_R_ is the respiratory rate in bpm. For example, when *f*_R_ is 10 bpm, we find τ_res,_ τ_rec_ < 3 s. As the human respiratory rate goes to 60 bpm in hyperpnea, a minimum τ_res,_ τ_rec_ of less than 0.5 s is desirable to monitor human respiration over the full bpm range. Thus, the development of humidity sensors with rapid response and recovery has been intensively studied. The number of publications relating to humidity sensors for respiratory monitoring has been increasing from 2017, as shown in Figure 3. 

### 2.3. Early Works of Respiratory Monitoring by Humidity Sensors

We first overview early works of monitoring respiratory signals using humidity sensors. In 1994, Muto et al. proposed an optical humidity sensor for monitoring breath condition [29]. They used an optical fiber with a humidity-sensitive cladding, which was made of a polymethyl methacrylate film and a dye showing fluorescence with water molecules. The sensor showed a response time of shorter than 1 s and was used for monitoring breathing behaviors with coughs. In 1997, Tatara and Tsuzaki used an electrical hygrometer with a membrane filter to observe medical respiratory patterns [30]. The response time of the sensor was evaluated to be 8 and 10 s with and without a membrane filter, respectively. The sensor was placed in front of a nasal orifice attached to the subject to detect the respiratory air. They experimentally observed respirations of an adult volunteer and compared the results with measurements from the humidity sensor and capnometry. In their system, the signal of the relative humidity preceded that of the CO_2_ concentration by ≈2 s. In the data outputted by the humidity sensor, rising/plateau and falling phases corresponded to expiration and inspiration of the subject, respectively. It should be noted that the recovery time of these humidity sensors was not short enough, and therefore, there was no plateau region in the inspiration phase of the humidity signals. A pause in inspiration was not clearly resolved in these experiments. 

In the field of sensor electronics, the microfabrication of humidity sensors is an effective strategy to improve their response and recovery times. Laville et al. proposed a capacitive humidity sensor using a polyimide layer as a pulmonary-function diagnostic microsystem in 2001 [31]. In this system, multiple physical sensors were equipped to observe the breathing conditions of patients suffering from chronic obstructive pulmonary disease and asthma. The sensor displayed a response and recovery time of 0.5 and 15 s, respectively, and demonstrated the observation of a single breath of air. In 2004, Kuban et al. measured respiratory rates by using a humidity sensor with electroplated interdigitated 50 μm line-and-spacing electrodes [32]. The sensor was fabricated by using microfabrication processes and a perfluorosulfonate ionomer (Nafion) as a solid electrolyte for the humidity-sensitive layer. The response time for an instant relative humidity (RH) change from 10 to 80% was 40 ms, allowing respiratory rates from 18 to 210 bpm to be clearly observed. Owing to the quick response, a plateau region in the inspiratory phase was resolved around an applied voltage of −0.05 V. Miyoshi et al. also microfabricated a flexible humidity sensor using a treated hydrophilic membrane for respiratory monitoring in 2009 [33]. The device showed a response time of 1 s and detected a breath rate of 9 bpm from a subject in a supine position. These were feasibility studies to experimentally detect breath air, and a further practical respiratory monitor had not been realized. These early works laid the groundwork for developing ultrafast humidity sensors with functional nanomaterials for respiratory monitoring in various situations. 

## 3. Types of Fast-Response Humidity Sensor

**Figure 4:**
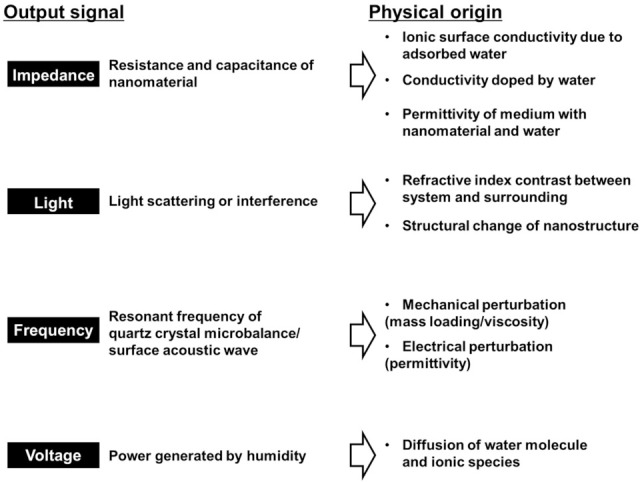
Four working principles of fast-response humidity sensors classified by physical origins of sensing.

In this review, we have classified the sensors by the types of output signals. Figure 4 shows four types of output signals in fast-response humidity sensors: impedance, light, frequency, and voltage, and the physical origin for the corresponding sensing mechanism. We summarize recent works to develop ultrafast humidity sensors and discuss the functional benefits from the viewpoint of humidity detection.

### 3.1. Impedance-Based Humidity Sensor

**Figure 5:**
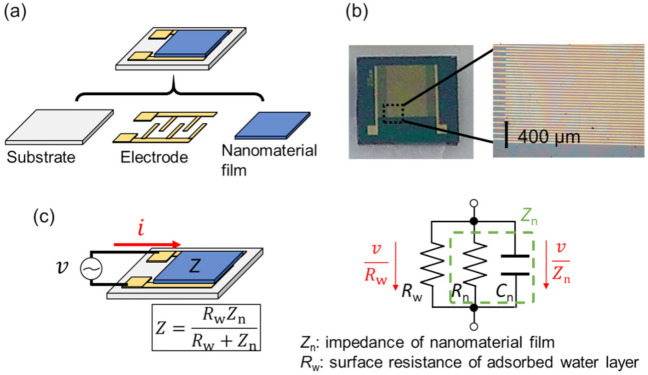
(**a**) Typical structure of an impedance-type humidity sensor. A humidity-sensitive nanomaterial film is coated over interdigitated electrodes. (**b**) Photo of a humidity sensor using a coated nanoparticle film and 20 μm line/spacing interdigitated electrodes on a thermally oxidized silicon substrate. (**c**) Equivalent electrical circuit of an impedance-type humidity sensor. *v*: voltage, *i*: current, *Z*: whole impedance (*v/i*), *R*_n_: resistance of nanomaterial film, *C*_n_: capacitance of nanomaterial film, and *R*_w_: surface resistance of adsorbed water layers on nanomaterial surface. *Z* corresponds to a parallel connection of *Z*_n_ and *R*_w_.

Reading the impedance of a sensor is a classical method to detect humidity in air [34,35,36]. In 1956, the fundamental invention of the humidity sensor based on electrolytic dissociation was first invented [34]. Currently, conventional humidity sensors using a polymer electrolyte have been installed in various humidity monitoring systems, including electrical appliances. The basic structure of an impedance-based humidity sensor consists of a humidity-sensitive nanomaterial film on electrodes, as shown in Figure 5a. The electrodes are typically interdigitated by microfabrication in order to reduce the total impedance. Figure 5b shows a sensor consisting of 20 μm line-and-spacing interdigitated electrodes and a coated nanoparticle film. A fringe due to the coated nanoparticle film appears over the electrode. 

An equivalent electrical circuit of the impedance-type sensor is shown in Figure 5c. Impedance-type sensors are characterized by two electrical parameters affected by humidity: resistance and capacitance. The total impedance *Z* is expressed as the impedance of the nanomaterial film *Z*_n_ and the surface resistance of the adsorbed water layers *R*_w_ in parallel. The film impedance is composed of resistive and capacitive components (*R*_n_ and *C*_n_). Therefore, we can use this structure as a humidity sensor if one of the three components (*R*_n_, *C*_n_, and *R*_w_) is dependent on humidity. It should be noted that an AC voltage is required to monitor the humidity dependence of the capacitive component as the capacitive impedance is inversely proportional to the applied frequency (1/*jωC*_n_, where ω is the angular frequency and *j* is an imaginary number). The humidity dependence of the resistive components can be monitored with a DC voltage, which is simple to implement with external devices such as through the input/output pins of microcontrollers.

**Figure 6:**
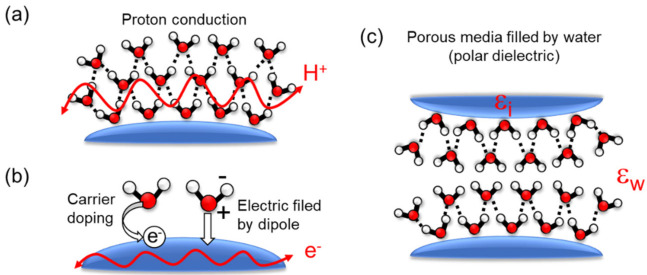
Physical origins of impedance modulation. (**a**) Proton ion conduction via water molecular chains. (**b**) Carrier modulation of semiconductor nanomaterials by water molecules. (**c**) Porous media filled by water molecules changing whole permittivity.

The humidity dependence of the impedance-type sensors can be described from the following physical origins. The humidity dependence of *R*_w_ is attributed to proton conduction through the water molecular layer on the surface. Proton hopping over the hydrogen bonds between water molecules due to an applied electric field is called the Grotthuss mechanism [37,38]. The current intensity from the Grotthuss mechanism is dependent on the thickness of the adsorbed water molecular layers and the thickness is dependent on the environmental humidity. A previous study regarding the phase of adsorbed water layers on silicon oxide indicates that it changes from solid-like at low humidity (0–30%) to liquid-like at high humidity (>60%) [39,40]. As water molecules can move more freely for exchanging protons in the liquid-like phase (i.e., both the number of charge carriers and their carrier mobility through the layers increase), *R*_w_ becomes smaller in higher humidity. This process is described in Figure 6a where the charge carrier is an ionic species. The mechanism is often used in electrolytic conductive materials (e.g., Nafion [32], polyelectrolytic polymer [41,42,43,44,45,46]) or insulating oxide materials with a hydrophilic surface [47,48,49,50,51,52,53]. Recently reported fast impedance-based humidity sensors rely on this mechanism, as shown later.

Alternatively, the conductance and electrical permittivity of the nanomaterial film can be directly modulated by the adsorption of water molecules, resulting in a humidity-dependent *R*_n_ and *C*_n_. Charge carriers doped from water molecules to the nanomaterial surface or an induced electric field by the water dipole are able to modulate the conductance (Figure 6b). In general, the conductance of a nanomaterial film *σ* is theoretically described as *σ* = *q_e_nμ*, where *q_e_* is the elementary charge, *n* is the carrier density of the film, and *μ* is the carrier mobility of the film. The carrier doping and the induced electric field can change *n* and *μ*, and thus, *σ* is modulated by the environmental humidity. This mechanism commonly appears in semiconductor and metal nanomaterial devices (e.g., graphene oxide [54,55,56,57], carbon nanotube/nanocoil [58,59], MXene nanosheet [60,61]) and is similarly used to detect other gases with metal oxide semiconductors, such as oxygen using SnO_2_ [62]. 

On the other hand, the capacitance of the nanomaterial is affected by the large dielectric permittivity of water (*ϵ*_w_ = 80). By filling pores in the nanomaterial film (*ϵ*_0_ = 1) with water molecules, as shown in Figure 6c, one can increase the effective capacitance of the film. A metal–dielectric–metal sandwiched structure is another candidate to increase the absolute value of capacitance. In this case, the top electrode needs to be carefully designed in order not to block the adsorption of water molecules into the insulator. For example, Itoh et al. demonstrated a sandwiched-type humidity sensor with a rise time of 0.15 s by using carbon nanotube film as a gas-permeable top electrode [63].

**Figure 7:**
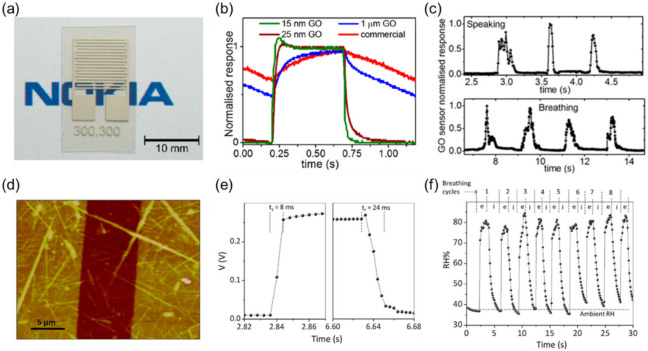
(**a**–**f**) Pioneering works to monitor breath air in real time by using fast-response impedance-type humidity sensors. (**a**) Photo of humidity sensor using a spray-coated graphene oxide (GO) film and silver electrodes on a polyethylene naphthalate substrate. (**b**) Normalized response of humidity sensors dependent on the thickness of graphene oxides. The response of a commercial humidity sensor is shown as a reference. (**c**) Detection of breath air from speaking and breathing by using an ultrathin graphene oxide humidity sensor. Courtesy: Adapted with permission from ref. [54]. Copyright 2003 American Chemical Society. (**d**) Atomic force microscope image of a few nanofibers over electrodes. (**e**) Response of humidity sensor using a few nanofibers. (**f**) Respiratory monitoring by using a nanofiber humidity sensor in real time. The notations, e and i, refer to the exhaling and inhaling of the subject, respectively. Courtesy: Adapted with permission from ref. [64]. Copyright 2014 Springer Nature.

Pioneering works to monitor breath air by using fast-response impedance-type humidity sensors were reported by Borini et al. in 2013 [54] and Mogera et al. in 2014 [64]. Borini et al. fabricated a flexible humidity sensor by using a coated graphene oxide and a silver interdigitated electrode on a polyethylene naphthalate substrate (Figure 7a). The graphene oxide film showed a hopping conduction of charge carriers through a network of overlapping films, which was modulated by the adsorption of water molecules. They found that the response and recovery of the sensor to the humidity change were dependent on the thickness of graphene oxide. The response and recovery times were evaluated by using a chopped humid airflow given to the sensor. According to their work, a 15 nm-thick film gave the shortest response and recovery times down to 30 ms (Figure 7b). The onset of the response was much sharper than that of a commercial capacitive humidity sensor based on a polymer thin film at that time. Through this property, they were able to detect breathing and speaking patterns with excellent temporal resolution, as shown in Figure 7c [54]. Mogera et al. used a few supramolecular nanofibers as the humidity-sensitive material across gold electrodes on a glass substrate (Figure 7d). A charge transfer between the nanofiber and water molecules induced a reduction in the fiber resistance at high RH. By using a voltage dividing circuit with a standard resistor, the response and recovery times due to humidity changes between 5 and 80%RH were measured to be 8 and 24 ms, respectively (Figure 7e). They demonstrated continuous monitoring of breathing cycles by measuring the humidity 3.5 cm away from the nose. Expiratory and inspiratory phases (noted as “e” and “i” respectively) of breathing were clearly distinguished from the response curves (Figure 7f) [64]. These studies paved the way for monitoring respiratory information by using humidity sensors with quick response/recovery times. The respiratory rate range usually covers up to 60 bpm, which translates to a 1 s breathing period. Thus, a fast-response sensor that exhibits response/recovery times of a few tens of milliseconds is quick enough to follow human respiration.

Recent works using impedance-type humidity sensors have been focusing on healthcare and medical applications. Zhen et al. obtained human respiratory information by using a wrinkled graphene-based humidity sensor [65]. This sensor was of a resistive type that used both mechanisms of ionic percolation through water networks and electrostatic interaction by the dipole moment of water molecules. The authors experimentally tested the sensor in several situations where a disturbance in human respiration was expected: physical activity, asthma, sudden arrest of breathing, coughing, and talking. Guan et al. reported on the trial to monitor respiration during sleep by humidity sensing [66]. The respiratory patterns in light and deep sleep of a voluntary subject were shown. A humidity sensor consisting of an insulating substrate with two copper electrodes was placed under the nose. The authors found that the amplitude of the current change decreases in deep sleep, indicating that breathing becomes weaker.

In addition to monitoring human respiration, Yan et al. demonstrated respiratory monitoring in rats by using a supramolecular ionic film-based humidity sensor [67]. Impedance-type humidity sensors can be miniaturized by using conventional microfabrication processes and, thus, it is possible to apply the sensor to small animals. Although the tidal volume of respiratory air is smaller (1–2 mL [68]) and the respiratory rate is faster (80–200 bpm for rats) than those of humans, the sensor successfully monitored the respiratory signals from rats. The humidity sensor monitored the increase in respiratory rates of an anesthetized rat from anesthetization to recovery. The change in rat respiration in response to drug injection was also monitored in real time by Wang et al. [68]. 

**Figure 8:**
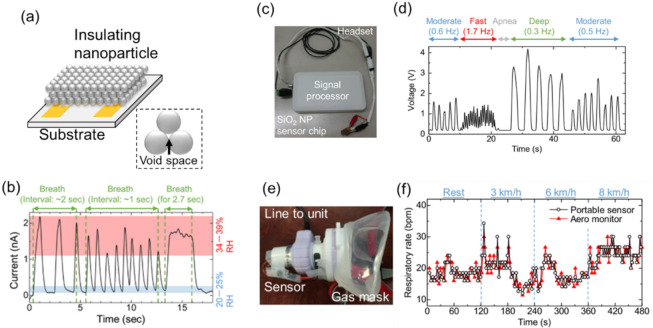
(**a**) Schematic of insulating nanoparticle film-based humidity sensor. (**b**) Respiratory monitoring by using the insulating nanoparticle-based sensor. Courtesy: Adapted with permission from ref. [70]. Copyright 2017 American Chemical Society. (**c**) A headset-type device using a nanoparticle film-based sensor. (**d**) Respiratory patterns observed by the monitor. The respiratory rate is intentionally controlled in this case. Courtesy: Adapted with permission from ref. [72] Copyright 2018 IEEE. (**e**) A nanoparticle film-based sensor attached on a gas mask of conventional respiratory monitoring system. (**f**) Change in respiratory rate during physical activity. The subject is at rest and running at 3, 6, and 8 km/h on a speed-controlled treadmill successively. Courtesy: Adapted with permission from ref. [73]. Copyright 2019 IEEE.

To realize a monitoring system of respiratory rates based on humidity, fast-response humidity sensors with an insulating nanoparticle film were investigated [16]. This sensor works as an ionic resistive-type humidity sensor. The surface of the insulating nanoparticle is hydrophilic, and the amount of adsorbed water molecules depends on environmental humidity. A film consisting of deposited nanoparticles includes void space between the nanoparticles, as shown in Figure 8a. If the size of the void space is appropriate, adsorption and desorption of water molecules effectively occur with changes in environmental humidity. If the void space is too small (around a few nanometers), adsorbed water molecules in the space are trapped by the capillary effect [69]. Figure 8b shows a typical response to breath air from a sensor using a surface-oxidized silicon nanoparticle film [70,71]. As counting the sharp response to breath air provides a respiratory rate in real time, a portable respiratory rate counter was proposed. Figure 8c shows a headset-type device consisting of a silicon dioxide (SiO_2_) nanoparticle-based sensor chip and a microcontroller with a Bluetooth connection [72]. The device can clearly monitor various breathing rates, from that of apnea (0 Hz) to 1.7 Hz = 102 bpm (Figure 8d). A preliminary test was carried out to compare the sensor with a conventional respiratory monitoring system. The fabricated sensor was attached to a gas mask, as shown in Figure 8e [73]. Respiratory rates were monitored for a subject at rest and running at 3, 6, and 8 km/h. As shown in Figure 8f, the insulating nanoparticle sensor (Portable sensor) measures the respiratory rates with a performance similar to the conventional system (Aero monitor). As a feasibility study, the signal from the humidity sensor was compared with the thoracic motion of breathing measured by an accelerometer on the neck [74]. From this experiment, the authors showed that monitoring human respiration with a fast-response humidity sensor is comparably robust to motion artefacts. 

### 3.2. Optical Humidity Sensor

#### 3.2.1. Optical Fiber Humidity Sensors

Optical fiber humidity sensors measure the humidity level by detecting changes in light propagation through a waveguide. Unlike their electronic counterparts, these sensors are unaffected by electromagnetic interference because the sensing components do not include electronic circuits [75,76]. Thus, they can be used as breathing monitors [77,78], ventilators, and other respiratory aids [79,80,81,82] for human patients in environments producing strong electromagnetic fields such as inside an MRI chamber. In addition, they are compact and lightweight, making them portable. The optical fiber humidity sensor generally consists of an optical core surrounded by a cladding. As light propagation in the fiber is governed by the refractive index contrast between the cladding and core, humidity-induced changes to the optical properties of the cladding modify the phase and intensity of the propagating light, detectable via interferometric methods or photosensitive devices, respectively. Fast response rates are achievable with nanostructured coatings within which water molecules can be rapidly absorbed or desorbed.

**Figure 9:**
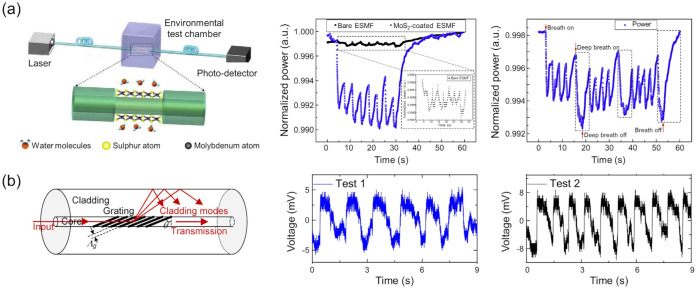
Demonstration of respiratory monitoring using optical fiber humidity sensors coated with nanomaterials. (**a**) Schematic of experimental setup for humidity measurement with an enlarged illustration of an MoS_2_-coated etched single-mode fiber (ESMF). The humidity responses from human breathing of the MoS_2_-coated ESMF sensor and a comparison to a bare ESMF sensor are shown in the middle and right panels, respectively [86]. Courtesy: Adapted with permission from ref. [86]. Copyright 2017 Elsevier. (**b**) Schematic of a large-angle tilted fiber grating (TFG) with graphene oxide deposited on the fiber surface. The bottom panel shows the humidity responses from normal (Test 1) and rapid (Test 2) breathing measurements [87]. Courtesy: Adapted with permission from ref. [87]. Copyright 2019 Elsevier.

Advances in optical waveguide design and humidity-sensitive material components have resulted in improved sensitivities, response times, target humidity ranges, accuracy, repeatability, durability, and cost-effectiveness. These have been comprehensively covered by previous reviews [75,83], including the various sensing mechanisms for humidity detection. Therefore, in this section, we limit our focus to studies relevant to respiratory monitoring, specifically those yielding sub-second response times and high sensitivities through the use of nanomaterials. Representative examples are shown in Figure 9. 

Recently, the use of a humidity-sensitive coating on a photonic crystal fiber (PCF) to modify the propagation of light in the mid-section of an optical fiber was proposed by Lopez-Torres et al. [84]. A 240 nm-thick poly(allylamine hydrochloride) (PAH) and poly(acrylic acid) (PAA) layer was applied on the PCF spliced between two single-mode fibers to create a humidity-sensitive section. Such a configuration represents a photonic crystal fiber interferometer (PCF-I), where light from the single-mode fiber is split to travel separately through the core and cladding of the PCF and recombine at the end of the PCF, resulting in interference. As the polymer coating swells and shrinks in response to humidity, light propagating along the cladding is altered, changing the interference pattern that translates to a sensitivity of 0.29 nm/%RH in the range of 20–75%RH. Owing to the thin polymer thickness, the sensor also exhibited average response and recovery times to human breath of 300 and 270 ms, respectively.

Changes in the transmitted power through an optical fiber decorated with 2D nanosheets have also been used to monitor the humidity. 2D nanosheets offer high surface-to-volume ratios that help the rapid adsorption of water molecules. When placed on polished or etched optical fibers, the 2D nanosheets couple with the evanescent field from the optical core, modifying the transmitted power through the fiber. Li et al. [85] prepared MoS_2_ nanosheets of 50 to 180 nm thickness on the polished part of an optical fiber. Both the average response and recovery times of the sensor were 85 ms. Similarly, Du et al. [86] reported on an etched single-mode fiber (ESMF) coated with MoS_2_ nanosheets. The variation in transmitted power with the MoS_2_-coated ESMF was found to be 14-fold higher than that of a bare ESMF. The authors also reported that the sensor achieved fast response and recovery times of 66 ms and 2.4 s, respectively, over the range of 20–80%RH. Although the exact mechanism behind the humidity responsivity from MoS_2_ needs further investigation, it is believed that the adsorbed water molecules change the charge carrier concentration of the nanosheets, modifying the polarizability and thus the effective complex refractive index of the nanosheets.

Graphene oxide is another class of 2D nanomaterials that exhibits strong humidity-responsivity. Jiang et al. [87] reported on an optical fiber sensor coated with graphene oxide, including a tilted fiber grating (TFG). Here, a tilted grating is introduced in the fiber core, which produces dual resonance peaks in the transmission spectrum that originate from the birefringence. By tracking the humidity-induced shift in the wavelength and intensity of one of the resonances, ultrafast response and recovery times of 42 ms and 115 ms, respectively, were found. This fast rate was attributed to the large surface area and surface-rich hydrophilic oxygen-containing groups on the graphene oxide layer. In addition, the sensor showed a response sensitivity of 18.5 pm/%RH in the range of 30–80%RH, suitable for detecting humidity variations in rapid breathing.

We note that most of the sensors above detect the transmitted signal through a fiber whose sidewall is functionalized by hygroscopic nanomaterials. Alternatively, one could also monitor the reflected signal from a fiber tip functionalized by humidity-responsive material. This approach was recently reported by Du et al. [88] where a 100 nm-thick plasmonic gold nanomembrane (Au NM) was attached to the fiber tip. The reflected spectrum from the fiber tip, displaying the plasmonic resonances of the Au NM, was modified with the condensation and evaporation of water on the NM. Nanoscale grooves and gaps in the Au NM were found to facilitate the capillary condensation of water, resulting in the fast rise and recovery times of 156 and 277 ms, respectively, when the RH changed from 11 to ~30%. Kang et al. [89] demonstrated an optical fiber whose tip was terminated with a 200 nm-thick film consisting of alternating layers of polymer and inorganic nanoclusters. On the surface of the polymer film, a thin layer of water condensed from warm vapor exhaled by a human. Interference patterns caused by light reflecting off the fiber tip/thin film, thin film/condensed water layer, and condensed water layer/air interfaces were used to detect the ambient humidity levels, where the condensed water layer thickness was assumed to vary with the humidity level. As the sensing mechanism depends on the water condensation and evaporation process, fast response and recovery times of less than 1 s were reported.

#### 3.2.2. Colorimetric Optical Humidity Sensors

**Figure 10:**
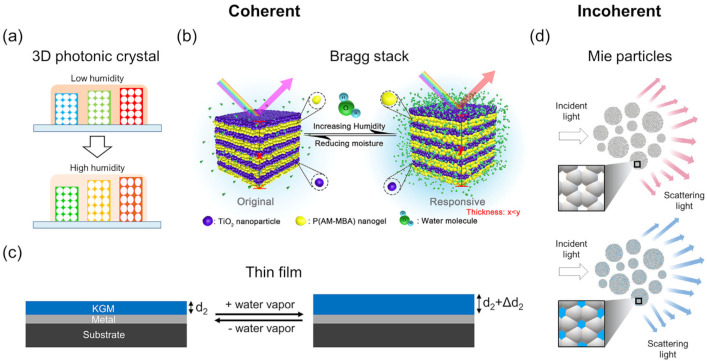
Schematic illustrating humidity-responsive structural colors from coherent (**a**–**c**) and incoherent (**d**) light interference. (**a**) A 3D photonic crystal composed of an inverse opal photonic gel in hydrophilic ionic liquid that swells and shrinks in response to humidity [92]. Courtesy: Adapted with permission from ref. [92]. Copyright 2018 MDPI. (**b**) Mesoporous 1D photonic crystal or Bragg stack, composed of alternating layers of nanogels and TiO_2_ nanoparticles [93]. Courtesy: Adapted with permission from ref. [93]. Copyright 2018 American Chemical Society. (**c**) A thin-film assembly of konjac glucomannan (KGM)–metal–substrate humidity sensor and KGM thickness changes as a result of humidity-induced swelling and shrinking [100]. Courtesy: Adapted with permission from ref. [100]. Copyright 2020 American Chemical Society. (**d**) Disordered assembly of mesoporous titania microspheres showing changes in scattered colors in response to humidity. The insets show models of the porous network inside the titania microsphere under dry (top) and humid (bottom) conditions [110]. Courtesy: Adapted with permission from ref. [110]. Copyright 2021 Royal Society of Chemistry.

Colorimetric humidity sensors are those that indicate the humidity level in color. The key benefit is that their response can be easily discerned visually or measured quantitatively using a photosensitive device. Although it is possible for the sensor to electrically measure the humidity and optically display color separately through humidity-sensitive material and optoelectronic platforms, less energy-intensive approaches that output signals directly from ambient light are available via structural colors. Structural colors are determined by the coherent or incoherent interference of light by judiciously arranged structured units, and can be modified via changes in refractive index contrast between units and the surroundings, structural parameters, or both. To this end, nanostructures play an important role as their sizes are commensurate with the wavelength of visible light, providing conditions amenable for light interference. If the material is humidity-responsive, that is, susceptible to changes in the optical constant or structure through interaction with water molecules, one can realize color variations that form the basis for colorimetric humidity sensing. Some of the most popular methods to create such structural colors from the coherent interference of light involve the ordered assembly of nanostructures in 1D (lamellar gratings), 2D (stacked films), and 3D (photonic crystals) or isolated nanostructures such as a single film (Figure 10). Assuming the surrounding is air, the interference condition for all these designs can be summarized by the Bragg equation (*mλ* = 2*dn*_eff_ sin*θ*), where *m* is an integer, *λ* is the light wavelength, *d* is the characteristic dimension, *n*_eff_ is the effective refractive index of the constituent material, and *θ* is the angle between the surface and incident light. It is apparent from the equation that a change in either the refractive index, structure, or both will shift the wavelength. As the incident angle also affects the colors, it is generally fixed to accurately assess changes in the refractive index or structure. 

Hydrogels are often used to produce a change in the structure as they swell or shrink in response to the input and output of moisture, respectively. However, the swelling and shrinking is temporally limited by the diffusion time of water molecules permeating throughout the gel and the deformation time of shifting chemically crosslinked polymer chains constituting the gel. Three-dimensional photonic crystals (PCs) made from opal hydrogels [90] and inverse opal hydrogels [91,92] showed varying volume and refractive indices in response to a change in RH from 20 to 100% that shifted diffracted colors up to 260 nm, in accordance to Bragg’s law but with limited response speeds from 9.4 to 36 s. Although 3DPCs offer large spectral changes, energy is expended in expanding and contracting the gel in three orthogonal directions. Faster speeds are, therefore, expected by reducing the dimension. 

**Figure 11:**
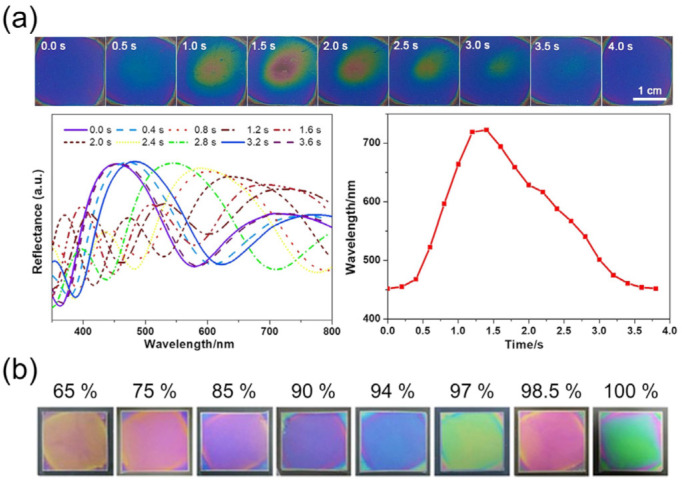
Colorimetric respiratory monitoring using structural color-based humidity sensors. (**a**) Reflectance spectra (left) and wavelength peak positions (right) of a P(AM-MBA)/TiO_2_ sensor under exposure to human exhalation. The inset on top shows photographs of the sensor taken at 0.5 s intervals [93]. Courtesy: Adapted with permission from ref. [93]. Copyright 2018 American Chemical Society. (**b**) Photographs showing colorimetric responses of the KGM humidity sensor at 65–100%RH [100]. Courtesy: Adapted with permission from ref. [100]. Copyright 2020 American Chemical Society.

Bragg stacks consisting of repeating units of high- and low-refractive-index layers, one of which is a hydrogel, swell and contract in one direction, that is, along the stack axis, decreasing the humidity-induced swelling and shrinking times considerably. Here, reflected light of each interface in the stack interferes coherently under conditions satisfying Bragg’s law for the stack: *mλ* = 2(*n_h_d_h_* + *n_l_d_l_*)sinθ, where *n_h_*, *d_h_*, *n_l_*, and *d_l_* are the refractive indices and thicknesses of the high- and low-index layers, respectively. Reports of such Bragg stacks composed of organic/inorganic hybrid materials [93,94], inorganic materials [95,96,97], and organic materials [98] have demonstrated controllable and bright structural colors. Kou et al. [93] recently reported the performance of a colorimetric Bragg stack humidity sensor responding to human breath, shown in Figure 11. The sensor was made of alternating layers of humidity-responsive poly(acrylamide-*N,N′*-methylene bis(acrylamide)) (P(AM-MBA)) nanogels and TiO_2_ nanoparticles forming textual mesopores. By optimizing the layer density, thickness, and stack number, the sensor exhibited a response time of ~0.5 s in response to humidity from respiration, marking an improvement over those from 3DPCs. Shifts in bright colors corresponding to wavelength differences of up to 242 nm were also found between 47 and 89%RH.

Further improvements in the response rates are possible by scaling down the multilayer stacks to a single layer because of smaller diffusion volumes and shorter adsorption-desorption paths for the water molecules. Structural colors from a single layer arise when the phase difference of light reflecting off the top and bottom interface of the film is an integer multiple of 2*π*. Practically, a single film is easier to prepare than multilayers comprising Bragg stacks.

Kim et al. used a 240 nm-thick partially sulfonated poly(styrene-*block*-methylbutylene) (PSS-b-PMB) block copolymer film [99] to demonstrate a colorimetric thin-film humidity sensor with response times below 2 s. The sensor displayed shifting colors up to 196 nm in wavelength from a 1.7-fold increase in film thickness in response to an increased RH from 20 to 90%. In addition to the optical response, a drop in electrical impedance due to proton transportation from one sulfonate group to another via water molecules provided a supplementary resistive mode for humidity measurement. Momtaz and Chen [100] also demonstrated a single-film colorimetric humidity sensor with large spectral shifts up to 385 nm through an increased RH from 85 to 100% that increased its thickness by 95.4%. The work is notable for the use of konjac glucomannan (KGM) as the thin film due to its nontoxic nature, highlighting its usability in healthcare. The rise and recovery times of the sensor were 1.5 and 3.5 s, respectively, for RH cycled between 43 and 91%. Based on similar principles, a colorimetric optical thin-film humidity sensor using a poly(diallyldimethylammonium)/poly(styrenesulfonate) (PDDA/PSS) polyelectrolyte coating on Si achieved a remarkable enhancement in the rise and recovery time of 35 and 95 ms, respectively [101]. The ultrafast rate was attributed to the use of a single film with nanoscale thickness (<400 nm) that enhances the diffusion of moisture through the film volume, and the electrostatic interactions between the PDDA and PSS chains that help the chains behave as mechanical springs for fast expansion and contraction. Aided by full-scale RH sensitivity, the sensors showed promise for tracking human breath patterns and enabling touchless control over other devices from the humidity near fingertips. 

In addition to polymers, graphene oxide is also a versatile humidity-responsive material. Graphene oxide decorated with hydrophilic oxygen-containing groups, such as hydroxyl, epoxy, and carboxylic groups, has been used for humidity sensing [54,102,103,104,105]. While most of these sensors are of the electrical type, a film of graphene oxide multilayers with an optical thickness that promotes the coherent interference of light has been reported to display humidity-responsive colors [105]. The insertion of water molecules between the graphene oxide multilayers contributes to the swelling of the film, redshifting the reflected light, while the removal shrinks the film, returning the color to its original state. The moisture absorption and desorption times were 0.25 and 1.2 s, respectively, for RH cycled between 50% and 90%, which are several times faster than those of most hydrogel-based optical sensors. The stability of the sensors in ambient atmosphere was maintained even after 1 year.

While the above demonstrations rely on the coherent interference of light, we note that structural colors can also be created from the incoherent interference of light. Photonic glass, as opposed to photonic crystals, composed of randomly arranged dielectric particles in bulk is known to display noniridescent colors [106,107,108]. In a similar spirit, a randomly arranged monolayer of polydisperse dielectric particles can also produce structural colors through the incoherent interference of Mie-scattered light from each particle [109]. If the particles are prepared to be mesoporous, of high-refractive index, and hydrophilic, water may diffuse throughout the volume of the particles, changing the effective refractive index and, hence, the scattered response. Unlike polymer films that require both moisture diffusion and volume swelling to display varying structural colors, these particles only require diffusion and offer more surface area per volume, resulting in ultrafast responses. Mohd-Noor et al. [110] reported such colorimetric humidity sensors from a single-to-few-layer coverage of polydisperse amorphous titania microspheres on glass prepared through simple spin-coating. In addition to displaying colorimetric changes, the titania particles demonstrated ultrafast rise and recovery times as short as 20 and 36 ms, respectively, upon exposure to RH cycles from ~20 to 90%. Limitations in the responsive RH scale up to 53% and lifetime up to several RH cycles were later dramatically improved up to ~85% and 90 cycles, respectively. This was achieved by increasing the mesopore sizes from <2 to ~7 nm and transitioning the amorphous titania particles into a polycrystalline phase that stabilizes the surface chemistry [111]. The enlarged pores provide two benefits: one is an increased ability to uptake water that increases the refractive index contrast with the surrounding and hence produces a larger change in scattering for improved optical responsivity, and the other is an enhanced diffusivity that improves the diffusion kinetics for faster response [112]. Employing this approach, the titania particles demonstrated faster rise and recovery times of 13 and 28 ms, respectively, compared to their predecessors, and were successfully used to precisely track vocal patterns with excellent reproducibility.

### 3.3. Frequency-Based Humidity Sensor

**Figure 12:**
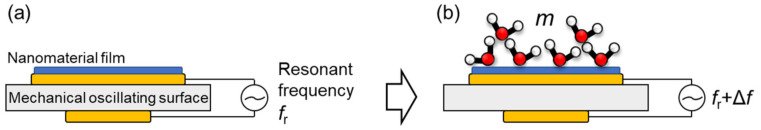
Physical origin of frequency-based humidity sensor using QCM. (**a**) Before and (**b**) after the adsorption of water molecules on QCM. The resonant frequency of the mechanical oscillating substrate is shifted by the adsorption. The advantage of this method is to evaluate the mass of adsorbed water molecules directly.

The resonant frequency of mechanical oscillators is affected by adsorbates on the surface. Figure 12 shows a schematic of a frequency-based humidity sensor using a quartz crystal microbalance (QCM). According to the Sauerbrey equation in QCM, the mass of adsorbates per unit area *m* is described as *m* = Δ*f*/*C*_f_, where Δ*f* is the shift in the resonant frequency *f*_r_ and *C*_f_ is a constant of the quartz crystal [113]. *C*_f_ is determined by *C*_f_ = 2*f*_r_^2^/*ρv*, where *ρ* is the density and *v* is the velocity of the shear wave propagating inside the crystal. The advantage of QCM is that it measures the mass of adsorbed water molecules quantitatively because Δ*f* is proportional to *m*. In the other sensing methods, it is required to calibrate the output of the humidity sensors for measuring the mass of adsorbates. Using a surface acoustic wave (SAW) resonator is another method to give a frequency output. Here, an acoustic wave propagates along the surface between emitting/receiving interdigitated electrodes. The transmission wave is scattered by the adsorbates of the surface due to a change in mass, viscosity, and electrical permittivity. Therefore, monitoring the transmission loss and the velocity of the wave can provide the amount of humidity in air.

**Figure 13:**
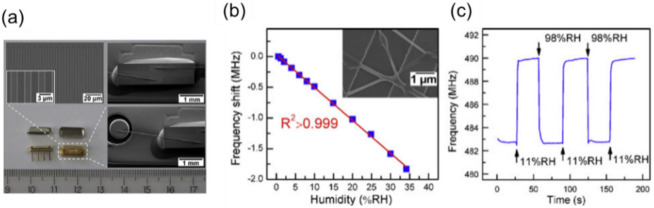
(**a**) Scanning electron microscope images of a surface-acoustic wave resonator. (**b**) Humidity dependence of resonant frequency shift. Inset: Electrospun polyaniline and polyvinyl butyral composite nanofibers. (**c**) Response and recovery time of the SAW sensor between 11 and 98%RH. Courtesy: Adapted with permission from ref. [116]. Copyright 2012 Elsevier.

QCMs have been classically used as a sensitive detector of surface adsorbates. For the rapid detection of humidity, Yao et al. evaluated the response of a QCM humidity sensor using a graphene oxide film with a thickness of 46 nm in 2011 [103]. The response and recovery times of the sensor were 18 and 12 s, respectively, for a changing RH between 11.3% and 97.3%. They found that a thinner graphene oxide film provides a shorter response time and smaller sensitivity (Δ*f* per %RH), attributed to the mass loads of adsorbed water molecules. Chen et al. recently reported a QCM-based humidity sensor for respiratory monitoring under the nose [114]. They used a bismuth oxychloride film having a hydrophilic surface as a humidity-sensitive layer. In their sensor, the logarithm of Δ*f* was proportional to the RH in ambient. The response and recovery times of the fabricated sensor were 5.2 and 4.5 s, respectively. Rimeika et al. proposed the possibility of a fast-response SAW humidity sensor with a hematoporphyrin film in 2009 [115]. The response and recovery times of the SAW transmission loss were 0.8 (from 13 to 26%RH) and 0.7 s (from 33 to 26%RH), respectively. They also showed a trial of respiratory rate monitoring using the SAW sensor. Lin et al. reported a SAW humidity sensor consisting of a SAW resonator (Figure 13a) and humidity-sensitive nanofibers [116]. Polyaniline and poly(vinyl buthyral) nanofibers were prepared on the resonator by electrospinning. The frequency shift of the resonance was linear with the environmental humidity (Figure 13b). The response and recovery times were estimated as 1 and 2 s, respectively, between 11 and 98%RH (Figure 13c).

### 3.4. Self-Powered Humidity Sensor

With the exception of structural color-based sensors, the above-mentioned sensors need a voltage supply to operate. Supplying electrical power to sensors is one of the largest problems for IoT applications in various situations. Recently, self-powered systems have been increasingly attracting attention for the realization of sustainable information societies. In the case of respiratory monitoring, a battery-less system is promising for realizing wearables with enhanced flexibility and comfort for users.

**Figure 14:**
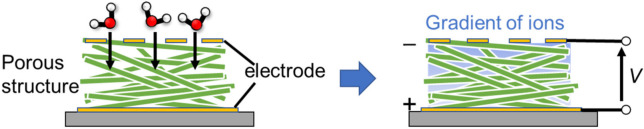
Typical mechanism of voltage generation from ambient humidity. A porous nanowire network charged in negative separate charged ions. Thus, a charge imbalance is formed and a potential difference is generated by humidity.

One solution is to harvest an electrical voltage from the humidity in breath air. Power generation from ambient humidity has been recently proposed [117,118,119]. Shen et al. demonstrated a self-powered wearable human breathing monitor by using designed TiO_2_ nanowire networks [120]. The device consists of a sandwiched structure with aluminum/TiO_2_ nanowire networks/indium tin oxide coated on a flexible poly(ethylene terephthalate) substrate. When the device is exposed to humid air, a gradient in the water concentration forms in the TiO_2_ networks. At the top side, the adsorbed water concentration is higher than that at the bottom side. This induces diffusion of ionic species related to water molecules (proton and hydronium ion) that subsequently generates a potential difference, as shown in Figure 14. Their moisture-enabled electricity generator (MEEG) provided an open-circuit voltage of 0.5 V and a power density of 4 μW/cm^2^ at 85%RH with which they realized battery-less respiratory monitoring. In this observation, a 1.2 × 1.2 cm-sized device was located 0.8 cm under the nose of the subject and generated a 20 mV output from a single breath. A respiratory rate up to 38 bpm was experimentally observed in the report. Li et al. reported respiratory monitoring by using a self-powered humidity sensor with a polypyrrole and melamine aerogel [121]. The sensor showed the response and recovery times of 1.1 and 4.5 s, respectively. The voltage generated by humidity in breath air was ≈100 mV.

Another solution is to drive a fast-response humidity sensor by another self-powered system. Zhang et al. reported a flexible humidity sensor driven by a triboelectric nanogenerator (TENG) [57]. TENG harvests electrical energy from mechanical friction between two materials [122]. As mechanical movements can be converted to a voltage, TENG has been recently used to monitor respiration from thoracic movements for wearables [26]. In the work of Zhang et al., the humidity sensor was of an ionic impedance type, based on SnO_2_ and reduced graphene oxide nanohybrid film. They used TENG to power the humidity sensor and monitored a respiratory rate up to 45 bpm.

**Figure 15:**
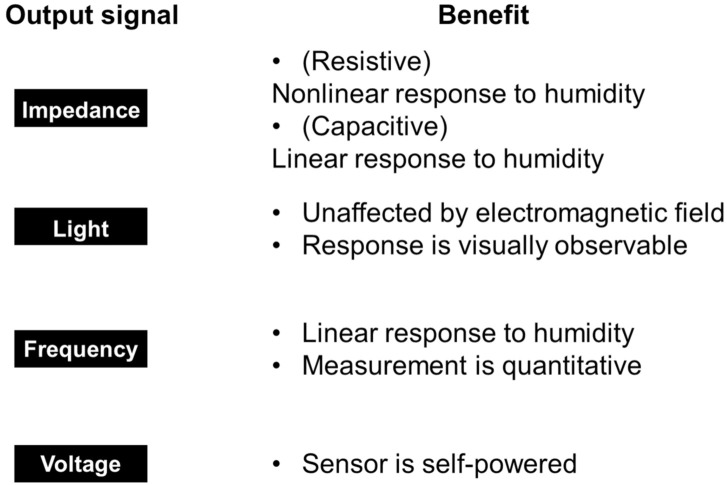
Benefits in each sensing mechanism.

As a summary of this section, we describe the benefits (advantages) of each type of device based on its output and humidity sensing mechanisms (Figure 15). For impedance-based outputs, there are resistive and capacitive types. The critical difference is the linearity of the output signal in response to changes in humidity. In resistive-type sensors, the impedance is typically nonlinear with respect to humidity change. The impedance changes by a few orders of magnitudes from low to high humidity [70,123,124,125,126]. Thus, resistive-type sensors are useful for quickly detecting high humidity. On the contrary, capacitive-type sensors respond linearly with respect to humidity change [127]. Thus, capacitive-type sensors are used to quantitatively measure the relative humidity with a simple electrical circuit. Optical sensors are unaffected by electromagnetic fields. Optical sensors based on structural colors provide the benefit of seeing the change in humidity with the naked eye. In addition, such sensors do not require a battery [110]. The benefit of frequency-output sensors is the ability to make quantitative measurements of the humidity content in air. QCM-based sensors are highly sensitive to adsorbates and respond linearly to the amount of the adsorbates in sub-monolayers. This mechanism can be superior for evaluating the amount of the adsorbates under humid air (i.e., how many molecular water layers are adsorbed at a certain relative humidity [43,60]). This evaluation is informative for considering in detail the other physical mechanisms of humidity sensing. For example, proton transport through molecular water layers is dependent on the thickness of the layers [39,40,128]. With voltage-output sensors, the benefit is that self-powered systems are achievable as a voltage is directly generated by a change in humidity such as in ambient air or human breath. This feature enables the development of wearable devices and IoT sensors without a separate power supply.

Despite the many advantages, there are also disadvantages to each type of device. For impedance and light-based outputs, one must precisely calibrate the device for volumetric measurements. For light-based reflective devices, it is obvious that information is inaccessible in dark environments. Frequency-type devices require large sizes commensurate with the resonant frequency (MHz to GHz), which hinder the portability of the device. The response of voltage-type outputs is relatively slow and further improvement in the conversion efficiency from humidity changes to electric power is required.

## 4. Response and Recovery of Reported Fast-Response Humidity Sensors

### 4.1. Comparison of Response/Recovery Time

**Figure 16:**
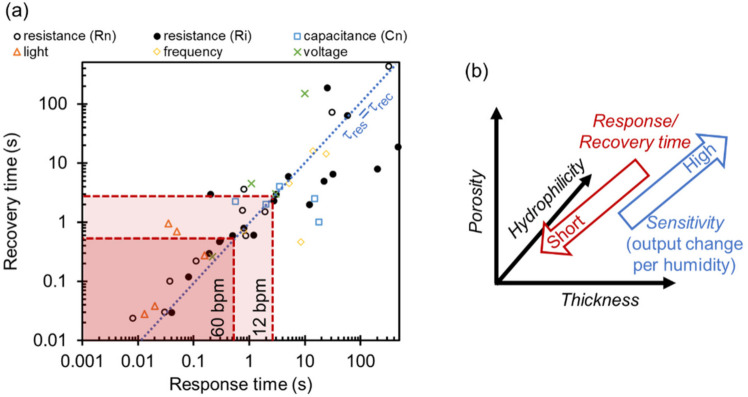
(**a**) Response (τ_res_) and recovery time (τ_rec_) of reported fast-response humidity sensors used for respiratory monitoring. A list of the literature is shown in Table S1 of Supplementary Materials. Rn, Cn: resistance and capacitance of nanomaterial film, respectively. Ri: resistance of adsorbed water layer. (**b**) Design strategy of a structure of nanomaterial films for fast-response humidity sensors.

Figure 16a plots the values of the response and recovery time from reported fast-response humidity sensors for respiratory monitoring. The dotted line represents the condition of equal response (*τ*_res_) and recovery time (*τ*_rec_). The reported values are dispersed from 100 to 0.01 s. Ultrafast response and recovery times shorter than 0.1 s are achieved by resistive-type [54,64,67], capacitive-type [129,130], and optical-type humidity sensors [82,110,111]. Frequency- and voltage-type sensors have relatively longer response and recovery times. We speculate the reason is that these sensors are highly sensitive to surface adsorbates and, therefore, a few molecular layers of water remaining on the surface may affect the output of the sensors. Ionic resistance and capacitance are modulated by the molecular layer thickness of water nonlinearly. For example, Grotthuss transport becomes more prominent as the thickness of molecular water layers increases [39]. This lower surface sensitivity possibly results in a faster response and recovery of the sensor output.

The red dashed lines represent the breathing periods of human respiration at the normal (12 bpm) and fastest (60 bpm) states. In order to track respiratory signals with sufficient temporal resolution, including breathing pose and depth, sensors having shorter response/recovery times than the threshold time are preferable. When a humidity sensor has a relatively longer response/recovery time than respiratory breathing periods, it is possible to only monitor respiratory rates but not respiratory rhythm and volume. To acquire only respiratory rates, we can use sensors with longer response/recovery times, as many reports have demonstrated. Güder et al. used digital filtering algorithms to extract respiratory signals from an impedance output of a paper-based humidity sensor [131]. An important issue for meaningful development is the standardization of humidity change used to evaluate the response and recovery times. As there is a no standardized method to evaluate the response and recovery times of humidity sensors, measurements have been carried out using separate protocols for changing the humidity. For example, intermittent humid gas [54,65,70,110,132], humid gas controlled by a humidifier [133,134,135], or saturated salt solutions [52,136,137] have been used to supply variable humidity to the sensors. Changes between lower humidity levels influence response and recovery times because the amount of adsorbed water molecules may affect the time for adsorption and desorption. An authorized standardization to evaluate response and recovery times is therefore required in the future to apply these sensors for industrial and commercial usages.

### 4.2. Design Strategy of Nanostructure to Achieve Ultrafast Response and Recovery

To shorten the response and recovery of the sensor, both adsorption and desorption processes need to be accelerated. In the field of gas sensors, a typical technique to enhance the desorption rate of adsorbates is to use a heater attached to the sensor. Recently, the room-temperature operation of gas sensors using nanomaterials was discussed for reducing the total power consumption of the sensor system [138]. In a similar manner, it is plausible to enhance the sensor response without a heater but by implementing a designed nanostructure for humidity detection. As summarized in Table S1, there are many reports of fast-response humidity sensors using designed nanomaterials. Based on previous literature, we propose a strategy to design humidity-sensitive nanomaterial films with fast-responses as follows (Figure 16b).

(A) Thickness

Humidity-sensitive nanomaterials are usually prepared by depositing the colloidal solution on a sensor substrate. The thickness of the materials can be controlled within a few tens of nanometers by well-designed coating processes, such as dip-coating, spin-coating, and spray-coating [139]. In agreement with previous studies on the thickness dependence [54,103], the response and recovery times are shorter in a thinner nanomaterial film. This dependence is reasonable because a deposited nanomaterial film is usually porous and the diffusion length of water molecules through the film is positively correlated with the thickness. It takes a shorter time to achieve thermal equilibrium between adsorbing/desorbing water molecules on a thinner nanomaterial film. This is the main reason why a single nanowire of supramolecular fibers [64] shows the fastest response reported to date, down to 8 ms, as shown in Figure 7e. One must note, however, that the amount of the adsorbates is also dependent on the thickness. The sensitivity to humidity change may be reduced with decreased thickness. Therefore, there is a general tradeoff between sensitivity and response speed.

(B) Porosity

The porosity of a nanomaterial film is also critical for determining the response and recovery of humidity sensors. According to the Kelvin law, a nanometer-scale pore generally captures adsorbates in the pore. The critical pressure of water vapor condensation strongly depends on the curvature of a liquid–vapor interface [69,140,141,142]. The relationship of relative humidity (*p*/*p*_0_: ratio of a partial vapor pressure *p* and a saturated vapor pressure *p*_0_) and pore curvature *r* is $\ln\left(\frac{p}{p_{0}}\right)=-\frac{2\gamma V_{m}}{rRT}$, where γ is the surface tension of liquid water, *V*_m_ is the molar volume of water, *R* is the gas constant, and *T* is the environmental temperature. A reduction in the critical pressure of condensation becomes obvious when the size of the nanopore is less than 100 nm. This phenomenon can make the response irreversible. Therefore, small nanopores of a few nanometers are not favorable for achieving fast response and recovery.

(C) Hydrophilicity

Water molecules interact strongly with a hydrophilic surface. As this can increase the output sensitivity to humidity change, most of the reported nanomaterials in fast-response humidity sensors are hydrophilic such as oxide nanomaterials (graphene oxide [54,102,103,105,143,144,145], silicon dioxide [16,39,47,52], titanium oxide [48,51,53,110,111,120,129]) and polymers [41,42,43,59,64,67,146]. Decreasing the adsorption energy of water molecules on the surface can be important for desorbing the molecules spontaneously when the environmental humidity decreases. As the surface interaction between water molecules and the surface can be modified with a hydrophobic chemical group, designing the nanomaterial surface to be hydrophilic possibly shortens the response time. A sufficient degree of hydrophobicity is beneficial to shorten the recovery time because adsorbed water molecules can be easily removed.

## 5. Benefits of Respiratory Monitoring Using Humidity Sensor

**Figure 17:**
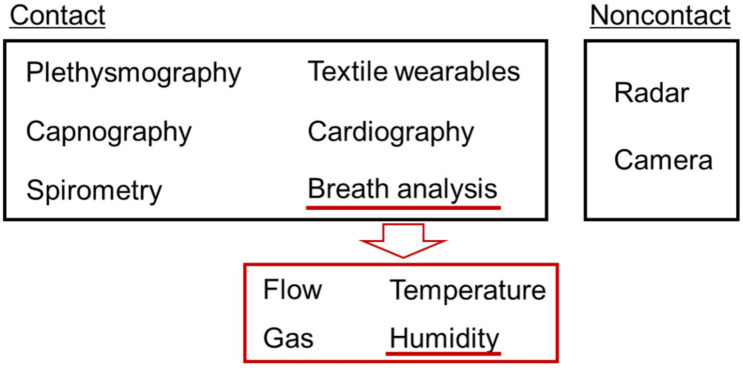
Conventional methods to monitor respiration activity.

In this section, we discuss the benefit to use fast-response humidity sensors for monitoring human respiration. Figure 17 shows conventional methods to monitor respiration activity. In clinical environments, the respiration of subjects is monitored with standard instruments such as bedside monitors, capnometers, and spirometers [13]. A bedside monitor operates by measuring the transthoracic impedance via electrode pads on the torso. This method provides respiratory rates and rhythms of the subject as the transthoracic impedance varies with body motion from breathing. A capnometer measures the amount of CO_2_ in expiratory air by using a cannula attached to the nose via tubes. A CO_2_ sensor is installed in the airway of the mechanical ventilation system such that the gas exchange of the patient during the ventilation can be monitored. CO_2_ serves as one of the fundamental regulators of cerebral blood flow, where hypercapnia (i.e., excessive CO_2_) causes the marked dilation of cerebral arteries and arterioles and increased blood flow, and hypocapnia (i.e., insufficient CO_2_) causes constriction and decreased blood flow. Thus, a capnometer is also an important tool for physiological studies on human behavior and brain activities. A spirometer uses an airflow sensor to measure the amount of breath air. The airflow sensor is typically a pressure sensor that calculates a volumetric flow rate from the pressure in the tube. By breathing air into the tube, the volumetric respiratory function of the lung (tidal volume, inspiratory/respiratory reserve volume) can be evaluated from the airflow signal. These methods are used for measuring the respiration of the subject at rest such as lying on a bed and sitting on a chair. A capnometer can also be used to measure respiration during exercise. 

In recent studies on respiratory monitoring during physical activity, contact-based methods have been popular. As Massaroni et al. summarized in 2019 [12], there are seven observables for the contact-based methods: respiratory airflow, respiratory sounds, air temperature, air humidity, air CO_2_, chest wall movements, and modulated cardiac activity. Currently, the most popular method is to measure chest wall movements with wearable sensor systems. The chest and abdomen synchronously move with inspiratory and expiratory phases during normal tidal breathing [1]. As such, textile-based wearables have been studied to obtain respiratory information in various situations [2,4,147,148,149,150,151,152,153]. Strain [154,155,156], acceleration [74,157,158,159,160], and capacitance [161] sensors on the body are commonly used for detecting body movements caused by breathing. However, motion artefacts can be a critical problem that affects the accuracy of the signals of the sensors. To reduce motion artefacts in the signal of breathing chest movements, wearables are usually contacted directly on or close to the torso.

Respiration monitoring using breath air does not require contact of the sensor to the torso. As the sensor signal is based on the physical properties of breath air: temperature, gas component, and humidity, the sensor is free from motion artefacts in principle. Thus, respiration during physical activity can be monitored accurately. In order not to interfere with the natural breathing of the subject, a comfortable and unobtrusive sensor system is recommended. Thermistors attached near the nose or the mouth have also been used to measure the temperature of breathing air.

Table 1 shows a comparison of respiration monitoring methods. The benefit of using a fast-response humidity sensor is its portability and unobtrusiveness. Under breath air, the sensor outputs a large signal due to the high humidity content. Thus, the signal-to-noise ratio is high as we can eliminate the need to install extra amplification components. By combining a humidity sensor with common portable IoT boards, we can carry out a pilot trial to monitor respiratory rates and rhythms with excellent versatility. Up until now, impedance outputs are frequently used for practical trials because the implementation into common IoT circuits is easier [65,72,131,162,163]. The portability and usability of devices are beneficial for us to monitor daily respiratory activities.

We note three reasons that hinder the practical use of respiratory monitoring with humidity sensors: an effect of the environment, a lack of volume measurements, and missing validation on a large number of human subjects. As the sensor signal is determined by the difference between humidity in breath air and ambient air, respiratory monitoring under saturated humidity (~100%RH) conditions is challenging. Ambient humidity and wind affect the respiratory monitoring by humidity sensors. Designing a device structure to suppress this environmental influence is required in the future. Currently, the volume of breath air is not quantitatively measured with a humidity sensor. Volumetric measurement is the next step for humidity-based respiratory observations. As Liu et al. commented [13], further validations on a large number of human subjects are needed to use humidity-based measurements for clinical purposes. Nicolo et al. summarized respiratory measurements for several purposes and discussed the need for measuring tidal volume, as well as respiratory rate [4]. The presence of breathing and the effect of cognitive load/physical effort do not always require volume measurements and, therefore, can be one of the first practical targets with portable humidity sensors. In future works, it is necessary to overcome these limitations for humidity sensors regarding respiratory monitoring.

## 6. Other Application for Physiological Monitoring

**Figure 18:**
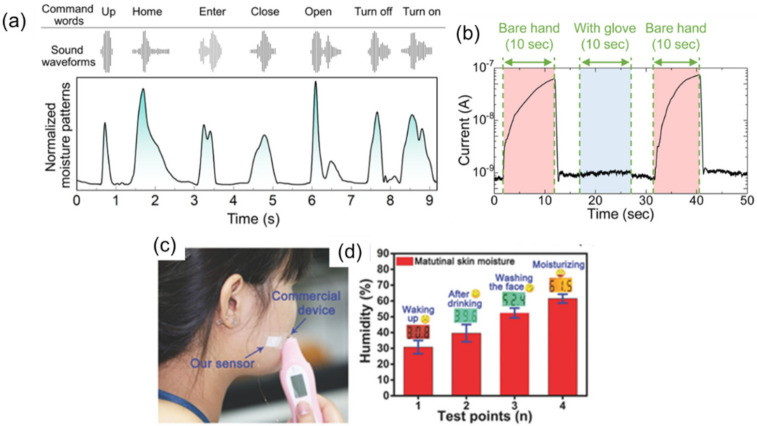
(**a**) Recognition of verbal commands by moisture patterns of exhaled breath air. Courtesy: Adapted with permission from ref. [111]. Copyright 2021 American Chemical Society. (**b**) Response with a bare hand vertically approaching and retracting away from the surface. A hand with a glove does not affect the current intensity. Courtesy: Adapted with permission from ref. [70] Copyright 2017 American Chemical Society. (**c**) A flexible humidity sensor applied to human skin to test skin moisture. (**d**) Variation in facial water contents under different conditions of the subject: waking up, drinking, washing face, and moisturizing face by moisturizer. Courtesy: Adapted with permission from ref. [170]. Copyright 2017 Wiley.

In addition to monitoring human respiration, fast-response humidity sensors can be used to monitor human physiological behaviors, as shown in this section [164]. Duan et al. summarized recent advancements in humidity sensors for human-body-related observations [17]. The recognition of verbal words has been reported from breath air [54,104,111,135,165]. Studies on phonation and voice patterns have been carried out by using strain sensors and pressure sensors on the neck of the subject [28]. Accompanying speech, breath air is simultaneously exhaled from the mouth. Therefore, it is possible to recognize spoken words through moisture patterns from the breath. Jarulertwathana et al. reported a comparison of moisture patterns from a fast-response humidity sensor and corresponding sound waveforms [111]. Large amplitudes of the sound waveforms correspond to the intensity of moisture patterns, as shown in Figure 18a. Accordingly, one can correlate certain enunciated parts of the words with the moisture content. This result may be helpful for interpreting speech from subjects with voice disorders. 

Another important application for fast-response humidity sensors is in monitoring water vapor from the skin. Transepidermal vapor transport is an important process describing water loss in human physiology [166,167]. As well as sweat secreted from sweat glands, water vaporization through the skin is a major source of water loss (transepidermal water loss (TEWL)) [166]. Several groups have recently demonstrated the detection of water vapor from the fingertip without contact [52,57,58,96,101,165,168,169,170]. The intensity of the sensor output is dependent on the height of the fingertip from the sensor surface. The trajectory of finger movements can be tracked by tracing water vapor from the fingertip, which can be used for noncontact user interface. Kano et al. presented the detection of a bare hand approaching a fast-response humidity sensor, as shown in Figure 18b [70].

TEWL is largely dependent on the skin condition. Rough skin accelerates the vaporization of water from the body surface. Yamamura et al. reported the relationship between skin disease and the amount of TEWL measured by a monochromatic refractometer [171]. The values of TEWL were 0.454 ± 0.087 mg/cm^2^/h for normal skin of young persons and 5.046 ± 0.882 mg/cm^2^/h for lesional skin of atopic patients. Li et al. reported the feasibility of studying skin moisture with a fast-response humidity sensor using a polymer gel electrolyte [170]. They compared the output of the sensor attached to the skin and water content obtained with a commercial skin monitor at the same time (Figure 18c). Over various activities, the sensor output (column in Figure 18d) showed a positive correlation with the skin moisture evaluated by the commercial sensor (digital data in Figure 18d).

## 7. Conclusions

In this review, we have overviewed recent developments on ultrafast humidity sensors made with functional nanomaterials for monitoring human respiration. Respiration is an important gauge of overall health, physical/mental activity, and emotions of human subjects. There are four main indicators: breath rate, rhythm, volume, and gas components. These indicators are informative for preparing precision healthcare solutions. As such, the development of advanced sensing systems is becoming an increasingly attractive field in materials science. We classify ultrafast humidity sensors according to four types of outputs: impedance, light, frequency, and voltage. For each type, we introduce the physical origin of humidity sensing and associated works marking pioneering and recent developments. The benefits using ultrafast humidity sensors are discussed for the application of respiratory monitoring. The response and recovery times of reported humidity sensors are compared, where the shortest times have reached 8 ms. Based on these reports, we discuss general design considerations for achieving fast response speeds, one of which is the tradeoff between response/recovery time and sensitivity. One of the key benefits of using humidity sensors in respiratory applications is that the sensors can be porfigureand noninvasive. Currently, ultrafast humidity sensors are used for observing respiratory rate and rhythm. Finally, we describe other areas of use besides respiration monitoring: voice recognition, noncontact user interface, and skin evaluation. So far, research in these areas using ultrafast humidity sensors has been limited to feasibility studies. It is important to carry out trials on a large number of human subjects to bring ultrafast humidity sensors closer to practical real-world applications.

## Figures and Tables

**Table 1:** Comparison of monitoring methods of respiration using breath air.

Breath Info	Breath Rate	Breath Rhythm	Breath Volume	Portability
Airflow	✓	✓	✓	
Temperature	✓	✓		
Gas	✓	✓	✓	
Humidity	✓	✓		✓

## Supplementary Materials

The following supporting information can be downloaded at: https://www.mdpi.com/article/10.3390/s22031251/s1, Table S1: Recent literature reporting fast-response humidity sensors for respiratory monitoring plotted in Figure 16a.
