# Supplementary material for: Respiratory Monitoring by Ultrafast Humidity Sensors with Nanomaterials: A Review
Source: Sensors (Basel). 2022 Feb 7;22(3):1251. doi: 10.3390/s22031251 (PMC8838830; doi:10.3390/s22031251)
Supplement: Supplementary file 1 [file sensors-22-01251-s001.zip › sensors-1572183-supplementary.pdf]

Supplementary Materials for

Respiratory Monitoring by Ultrafast Humidity Sensors with Nanomaterials: A Review

Shinya Kano[1,2,*], Nutpaphat Jarulertwathana[3], Syazwani Mohd-Noor[3], Jerome K. Hyun[3], Ryota Asahara[4], and Harutaka Mekaru[1,2]

[1] Human Augmentation Research Center, National Institute of Advanced Industrial Science and Technology (AIST), Kashiwa 277-8882, Japan
[2] Sensing System Research Center, National Institute of Advanced Industrial Science and Technology (AIST), Tsukuba 305-8563, Japan
[3] Department of Chemistry and Nanoscience, Ewha Womans University, Seoul 03760, Republic of Korea
[4] Human Informatics and Interaction Research Institute, National Institute of Advanced Industrial Science and Technology (AIST), Tsukuba 305-8566, Japan
* Correspondence: shinya-kano@aist.go.jp

Table S1. Recent literature reporting fast-response humidity sensors for respiratory monitoring plotted in Figure 16(a). RH: relative humidity. R_n: resistance of nanomaterial film. C_n: capacitance of nanomaterial film. R_w: ionic resistance of adsorbed water molecular layer.

Materials	Type	Response time (s)	Recovery time (s)	RH change	Reference
**graphene oxide**	resistance (R_n)	0.03	0.03	40-75%	S. Borini, ACS Sens. 2013.[1]
**supramolecular nanofibers**	resistance (R_n)	0.008	0.024	5-80%	U. Mogera, Sci. Rep. 2014.[2]
**supramolecular ionic material**	resistance (R_n)	0.037	0.1	0-11%	H. Yan, Anal. Chem. 2017.[3]
**carbon nanocoil**	resistance (R_n)	1.9	1.5	0-16%	J. Wu, ACS Appl. Mater. Interfaces 2019. [4]
**MXene nanosheet/polyelectrolyte multilayer**	resistance (R_n)	0.11	0.22	20-40%	H. An, ACS Appl. Nano Mater. 2019.[5]
**nanoporous cellulose nanofiber/carbon nanotube/paper fiber**	resistance (R_n)	321	435	11-95%	P. Zhu, ACS Appl. Mater. Interfaces 2020.[6]
**PEDOT:PSS micron line**	resistance (R_n)	0.86	0.59	42-69%	G. Wang, RSC Advances 2020.[7]
**tunnel-cracked nickel@polyurethane sponge**	resistance (R_n)	0.8	3.6	10-70%	Q. Wang, Sens. Act. B 2021.[8]
**MXene/chitosan-quercerin multilayer**	resistance (R_n)	0.75	1.6	1-90%	X. Li, Sens. Act. B 2021.[9]
**nafion**	resistance (R_i)	0.04	0.03	0-98%	P. Kuban, Anal. Chem. 2004.[10]
**porous SnO_2**	resistance (R_i)	200	8	2-97%	V. Solanki, ACS Appl. Mater. Interfaces 2017.[11]
**silicon nanocrystal**	resistance (R_i)	12	2	20-95%	S. Kano, ACS Sens. 2017.[12]
**WS_2 nanosheet**	resistance (R_i)	5	6	35-40%	H. Guo, Nanoscale 2017.[13]
**silica nanoparticle**	resistance (R_i)	31.4	6.5	30-84%	S. Kano, ACS Sust. Chem. Eng. 2018.[14]
**$NiPS_3$ nanosheet**	resistance (R_i)	3	3	0-32%	R. N. Jenjeti, J. Mater. Chem. A 2019.[15]
**Ti_3C_2/Ag hybrid composite**	resistance (R_i)	0.08	0.12	5-95%	N. Li, Nanoscale 2019.[16]
**cellulose (paper)**	resistance (R_i)	472	19	7.2-91.5%	Z. Duan, ACS Appl. Mater. Interfaces 2019.[17]
**polyelectrolyte (PDMS, PPDS)**	resistance (R_i)	0.29	0.47	33-95%	J. Dai, ACS Appl. Mater. Interfaces 2019.[18]

**MoO_3 nanosheet**	resistance (R_i)	0.3	0.5	0-40%	J. Yang, Small 2019.[19]
**gel polymer (MPOSS-PIL)**	resistance (R_i)	0.19	0.3	11-95%	J. Dai, Sens. Act. B 2020.[20]
**polyacrylic acid**	resistance (R_i)	1.2	0.6	11-95%	J. Dai, Sens. Act. B 2020.[21]
**50-nm silica nanoparticle**	resistance (R_i)	2.8	2.3	30-70%	S. Kano, Sensors 2020.[22]
**SnS_2 nanoflower/reduced graphene oxide**	resistance (R_i)	4	3	0-33%	D. Zhang, Nano Energy 2020.[23]
**Au nanoparticle modified TiO_2**	resistance (R_i)	58	64	11-95%	S. Yu, Ceramics International 2021.[24]
**glycidyl trimethyl ammonium chloride modified cellulose**	resistance (R_i)	25	188	11-95%	X. Guan, Sens. Act. B 2021.[25]
**coolmax/graphene oxide functionalized textile**	resistance (R_i)	0.5	0.6	45-80%	L. Xu, Chem. Eng. J. 2021.[26]
**graphite carbon nitride/zinc oxide**	resistance (R_i)	22	5	11-95%	S. Yu, Sens. Act. B 2021.[27]
**Pb/HNb_3O_8**	resistance (R_i)	0.2	3	30-96%	Y. Lu, Nanoscale Horiz. 2021.[28]
**Fe_2O_3-reduced graphene oxide nanohybrid**	resistance (R_i)	0.8	0.8	5-97%	A. Kumar, J. Mater. Chem. C 2021.[29]
**SnS_2 nanoflower/Zn_2SnO_4 hollow sphere**	capacitance (C_n)	18	1	0-97%	D. Zhang ACS AMI 2018.[30]
**polyester fiber**	capacitance (C_n)	3.5	4	6-33%	L. Ma, Adv. Func. Mat. 2019.[31]
**In_2O_3 nanocube/graphene oxide nanosheet**	capacitance (C_n)	15	2.5	0-43%	B. Li, Sens Act B 2019.[32]
**$CsPb_2Br_5/BaTiO_3$**	capacitance (C_n)	2	2	30-80%	M.-Y. Cho, ACS Appl. Mater. Interfaces 2021.[33]
**reduced graphene oxide/WS_2 heterojunction**	capacitance (C_n)	0.56	2.26	12.5-85.2%	L. Zhang, Sens. Act. B 2021.[34]
**PDDA/PSS layers**	light	0.035	0.95	45-90%	L. Yu, Mater. Horiz. 2017.[35]
**agarose coated fiber**	light	0.05	0.7	60-90%	J. Mathew, Sens. Act. A 2012.[36]
**titania microsphere**	light	0.02	0.038	20-90%	S. Mohd-Noor J. Mater. Chem. A 2019.[37]
**gold nanomembrane with optical fiber**	light	0.156	0.277	11-30%	B. Du, Opt. Lett. 2020.[38]

**yolk-shell titania microsphere**	light	0.013	0.028	20-90%	N. Jarulertwathana, ACS Appl. Mat. Interfaces 2021.[39]
**In_2O_3 quantum dots**	frequency (QCM)	14	16	0-75.3%	H. Kan, RSC Advances 2019.[40]
**bismuth oxychloride**	frequency (QCM)	5.2	4.5	11.3-84.3%	Q. Chen, ACS Omega 2020.[41]
**gel polymer (MPOSS-PIL)**	frequency (QCM)	8.5	0.46	11-95%	J. Dai, Sens. Act. B 2020.[20]
**hematoporphyrin-lithium niobate**	frequency (SAW)	0.8	0.7	13-26%, 33-26%	R. Rimeika, Sens. Act. B 2009.[42]
**graphene/polyvinyl alcohol/SiO_2**	frequency (SAW)	24	14.4	0-70%	Y. Su, Sens. Act. B 2020.[43]
**graphene oxide with oxygen-containing group gradient**	voltage	3	3	variation=30 %	F. Zhao, Adv. Mater. 2015.[44]
**TiO_2 nanowire network**	voltage	10	150	10-85%	D. Shen, Adv. Mater. 2018.[45]
**porous polydopamine with hydroxyl group gradient**	voltage	0.22	0.26	5-55%	L. Li, Nano Lett. 2019.[46]
**polypyrrole/melamine aerogel**	voltage	1.1	4.5	50-75%	X. Li, IEEE Sens. 2021.[47]

1. Borini, S.; White, R.; Wei, D.; Astley, M.; Haque, S.; Spigone, E.; Harris, N.; Kivioja, J.; Ryhänen, T. Ultrafast Graphene Oxide Humidity Sensors. *ACS Nano* **2013**, *7*, 11166–11173, doi:10.1021/nn404889b.
2. Mogera, U.; Sagade, A.A.; George, S.J.; Kulkarni, G.U. Ultrafast response humidity sensor using supramolecular nanofibre and its application in monitoring breath humidity and flow. *Sci. Rep.* **2014**, *4*, 4103, doi:10.1038/srep04103.
3. Yan, H.; Zhang, L.; Yu, P.; Mao, L. Sensitive and Fast Humidity Sensor Based on A Redox Conducting Supramolecular Ionic Material for Respiration Monitoring. *Anal. Chem.* **2017**, *89*, 996–1001, doi:10.1021/acs.analchem.6b04350.
4. Wu, J.; Sun, Y.-M.; Wu, Z.; Li, X.; Wang, N.; Tao, K.; Wang, G.P. Carbon Nanocoil-Based Fast-Response and Flexible Humidity Sensor for Multifunctional Applications. *ACS Appl. Mater. Interfaces* **2019**, *11*, 4242–4251, doi:10.1021/acsami.8b18599.
5. An, H.; Habib, T.; Shah, S.; Gao, H.; Patel, A.; Echols, I.; Zhao, X.; Radovic, M.; Green, M.J.; Lutkenhaus, J.L. Water Sorption in MXene/Polyelectrolyte Multilayers for Ultrafast Humidity Sensing. *ACS Appl. Nano Mater.* **2019**, *2*, 948–955, doi:10.1021/acsanm.8b02265.
6. Zhu, P.; Ou, H.; Kuang, Y.; Hao, L.; Diao, J.; Chen, G. Cellulose Nanofiber/Carbon Nanotube Dual Network-Enabled Humidity Sensor with High Sensitivity and Durability. *ACS Appl. Mater. Interfaces* **2020**, *12*, 33229–33238, doi:10.1021/acsami.0c07995.

7. Wang, G.; Zhang, Y.; Yang, H.; Wang, W.; Dai, Y.Z.; Niu, L.G.; Lv, C.; Xia, H.; Liu, T. Fast-response humidity sensor based on laser printing for respiration monitoring. *RSC Adv.* **2020**, *10*, 8910–8916, doi:10.1039/c9ra10409g.
8. Wang, Q.; Tong, J.; Wang, N.; Chen, S.; Sheng, B. Humidity sensor of tunnel-cracked nickel@polyurethane sponge for respiratory and perspiration sensing. *Sensors Actuators, B Chem.* **2021**, *330*, 129322, doi:10.1016/j.snb.2020.129322.
9. Li, X.; Lu, Y.; Shi, Z.; Liu, G.; Xu, G.; An, Z.; Xing, H.; Chen, Q.; Han, R.P.S.; Liu, Q. Onion-inspired MXene/chitosan-quercetin multilayers: Enhanced response to H2O molecules for wearable human physiological monitoring. *Sensors Actuators, B Chem.* **2021**, *329*, 129209, doi:10.1016/j.snb.2020.129209.
10. Kuban, P.; Berg, J.M.; Dasgupta, P.K. Durable Microfabricated High-Speed Humidity Sensors. *Anal. Chem.* **2004**, *76*, 2561–2567, doi:10.1021/ac0355451.
11. Solanki, V.; Krupanidhi, S.B.; Nanda, K.K. Sequential Elemental Dealloying Approach for the Fabrication of Porous Metal Oxides and Chemiresistive Sensors Thereof for Electronic Listening. *ACS Appl. Mater. Interfaces* **2017**, *9*, 41428–41434, doi:10.1021/acsami.7b12127.
12. Kano, S.; Kim, K.; Fujii, M. Fast-Response and Flexible Nanocrystal-Based Humidity Sensor for Monitoring Human Respiration and Water Evaporation on Skin. *ACS Sensors* **2017**, *2*, 828–833, doi:10.1021/acssensors.7b00199.
13. Guo, H.; Lan, C.; Zhou, Z.; Sun, P.; Wei, D.; Li, C. Transparent, flexible, and stretchable WS 2 based humidity sensors for electronic skin. *Nanoscale* **2017**, *9*, 6246–6253, doi:10.1039/C7NR01016H.
14. Kano, S.; Fujii, M. All-Painting Process To Produce Respiration Sensor Using Humidity-Sensitive Nanoparticle Film and Graphite Trace. *ACS Sustain. Chem. Eng.* **2018**, *6*, 12217–12223, doi:10.1021/acssuschemeng.8b02550.
15. Jenjeti, R.N.; Kumar, R.; Sampath, S. Two-dimensional, few-layer NiPS 3 for flexible humidity sensor with high selectivity. *J. Mater. Chem. A* **2019**, *7*, 14545–14551, doi:10.1039/C9TA03214B.
16. Li, N.; Jiang, Y.; Xiao, Y.; Meng, B.; Xing, C.; Zhang, H.; Peng, Z. A fully inkjet-printed transparent humidity sensor based on a Ti3C2/Ag hybrid for touchless sensing of finger motion. *Nanoscale* **2019**, *11*, 21522–21531, doi:10.1039/c9nr06751e.
17. Duan, Z.; Jiang, Y.; Yan, M.; Wang, S.; Yuan, Z.; Zhao, Q.; Sun, P.; Xie, G.; Du, X.; Tai, H. Facile, Flexible, Cost-Saving, and Environment-Friendly Paper-Based Humidity Sensor for Multifunctional Applications. *ACS Appl. Mater. Interfaces* **2019**, *11*, 21840–21849, doi:10.1021/acsami.9b05709.
18. Dai, J.; Zhao, H.; Lin, X.; Liu, S.; Liu, Y.; Liu, X.; Fei, T.; Zhang, T. Ultrafast Response Polyelectrolyte Humidity Sensor for Respiration Monitoring. *ACS Appl. Mater. Interfaces* **2019**, doi:10.1021/acsami.8b18904.
19. Yang, J.; Shi, R.; Lou, Z.; Chai, R.; Jiang, K.; Shen, G. Flexible Smart Noncontact Control Systems with Ultrasensitive Humidity Sensors. *Small* **2019**, *15*, 1902801, doi:10.1002/smll.201902801.
20. Dai, J.; Zhao, H.; Lin, X.; Liu, S.; Fei, T.; Zhang, T. Design strategy for ultrafast-response humidity sensors based on gel polymer electrolytes and application for detecting respiration. *Sensors Actuators B Chem.* **2020**, *304*, 127270, doi:10.1016/j.snb.2019.127270.

21. Dai, J.; Guan, X.; Zhao, H.; Liu, S.; Fei, T.; Zhang, T. In Situ Preparation of Porous Humidity Sensitive Composite via a One-Stone-Two-Birds Strategy. *Sensors Actuators, B Chem.* **2020**, *316*, 128159, doi:10.1016/j.snb.2020.128159.
22. Kano, S.; Mekaru, H. Nonporous Inorganic Nanoparticle-Based Humidity Sensor: Evaluation of Humidity Hysteresis and Response Time. *Sensors* **2020**, *20*, 3858, doi:10.3390/s20143858.
23. Zhang, D.; Xu, Z.; Yang, Z.; Song, X. High-performance flexible self-powered tin disulfide nanoflowers/reduced graphene oxide nanohybrid-based humidity sensor driven by triboelectric nanogenerator. *Nano Energy* **2020**, *67*, 104251, doi:10.1016/j.nanoen.2019.104251.
24. Yu, S.; Zhang, H.; Zhang, J. Synthesis of high response gold/titanium dioxide humidity sensor and its application in human respiration. *Ceram. Int.* **2021**, *47*, 30880–30887, doi:10.1016/j.ceramint.2021.07.270.
25. Guan, X.; Hou, Z.; Wu, K.; Zhao, H.; Liu, S.; Fei, T.; Zhang, T. Flexible humidity sensor based on modified cellulose paper. *Sensors Actuators B Chem.* **2021**, *339*, 129879, doi:10.1016/j.snb.2021.129879.
26. Xu, L.; Zhai, H.; Chen, X.; Liu, Y.; Wang, M.; Liu, Z.; Umar, M.; Ji, C.; Chen, Z.; Jin, L.; et al. Coolmax/graphene-oxide functionalized textile humidity sensor with ultrafast response for human activities monitoring. *Chem. Eng. J.* **2021**, *412*, doi:10.1016/j.cej.2021.128639.
27. Yu, S.; Chen, C.; Zhang, H.; Zhang, J.; Liu, J. Design of high sensitivity graphite carbon nitride/zinc oxide humidity sensor for breath detection. *Sensors Actuators, B Chem.* **2021**, *332*, 129536, doi:10.1016/j.snb.2021.129536.
28. Lu, Y.; Xu, K.; Yang, M.Q.; Tang, S.Y.; Yang, T.Y.; Fujita, Y.; Honda, S.; Arie, T.; Akita, S.; Chueh, Y.L.; et al. Highly stable Pd/HNb3O8-based flexible humidity sensor for perdurable wireless wearable applications. *Nanoscale Horizons* **2021**, *6*, 260–270, doi:10.1039/d0nh00594k.
29. Kumar, A.; Kumar, A.; Varma, G.D. Ultrafast resistive-type γ-Fe 2 O 3 –rGO nanohybrid-based humidity sensor – a respiratory monitoring tool . *J. Mater. Chem. C* **2021**, *9*, 8002–8010, doi:10.1039/d1tc01217g.
30. Zhang, D.; Zong, X.; Wu, Z.; Zhang, Y. Hierarchical Self-Assembled SnS 2 Nanoflower/Zn 2 SnO 4 Hollow Sphere Nanohybrid for Humidity-Sensing Applications. *ACS Appl. Mater. Interfaces* **2018**, *10*, 32631–32639, doi:10.1021/acsami.8b08493.
31. Ma, L.; Wu, R.; Patil, A.; Zhu, S.; Meng, Z.; Meng, H.; Hou, C.; Zhang, Y.; Liu, Q.; Yu, R.; et al. Full-Textile Wireless Flexible Humidity Sensor for Human Physiological Monitoring. *Adv. Funct. Mater.* **2019**, *29*, 1–9, doi:10.1002/adfm.201904549.
32. Li, B.; Tian, Q.; Su, H.; Wang, X.; Wang, T.; Zhang, D. High sensitivity protable capacitive humidity sensor based on In2O3 nanocubes-decorated GO nanosheets and its wearable application in respiration detection. *Sensors Actuators, B Chem.* **2019**, *299*, 126973, doi:10.1016/j.snb.2019.126973.
33. Cho, M.Y.; Kim, I.S.; Kim, S.H.; Park, C.; Kim, N.Y.; Kim, S.W.; Kim, S.; Oh, J.M. Unique Noncontact Monitoring of Human Respiration and Sweat Evaporation Using a CsPb2Br5-Based Sensor. *ACS Appl. Mater. Interfaces* **2021**, *13*, 5602–5613, doi:10.1021/acsami.0c21097.

34. Zhang, L.; Tan, Q.; Wang, Y.; Fan, Z.; Lin, L.; Zhang, W.; Xiong, J. Wirelessly powered multi-functional wearable humidity sensor based on RGO-WS2 heterojunctions. *Sensors Actuators, B Chem.* **2021**, *329*, 129077, doi:10.1016/j.snb.2020.129077.
35. Yu, L.; Xu, H.; Monro, T.M.; Lancaster, D.G.; Xie, Y.; Zeng, H.; Chen, G.Y.; Liu, X. Ultrafast colorimetric humidity-sensitive polyelectrolyte coating for touchless control. *Mater. Horizons* **2017**, *4*, 72–82, doi:10.1039/c6mh00317f.
36. Mathew, J.; Semenova, Y.; Farrell, G. A fiber bend based humidity sensor with a wide linear range and fast measurement speed. *Sensors Actuators, A Phys.* **2012**, *174*, 47–51, doi:10.1016/j.sna.2011.11.029.
37. Mohd-Noor, S.; Jang, H.; Baek, K.; Pei, Y.-R.; Alam, A.-M.; Kim, Y.H.; Kim, I.S.; Choy, J.-H.; Hyun, J.K. Ultrafast humidity-responsive structural colors from disordered nanoporous titania microspheres. *J. Mater. Chem. A* **2019**, *7*, 10561–10571, doi:10.1039/C9TA01394F.
38. Du, B.; Yang, D.; Ruan, Y.; Jia, P.; Ebendorff-Heidepriem, H. Compact plasmonic fiber tip for sensitive and fast humidity and human breath monitoring. *Opt. Lett.* **2020**, *45*, 985, doi:10.1364/OL.381085.
39. Jarulertwathana, N.; Mohd-Noor, S.; Hyun, J.K. Mesoporous Solid and Yolk-Shell Titania Microspheres as Touchless Colorimetric Sensors with High Responsivity and Ultrashort Response Times. *ACS Appl. Mater. Interfaces* **2021**, *13*, 44786–44796, doi:10.1021/acsami.1c12514.
40. Kan, H.; Li, M.; Li, H.; Li, C.; Zhou, J.; Fu, C.; Luo, J.; Fu, Y. A novel quartz-crystal microbalance humidity sensor based on solution-processible indium oxide quantum dots. *RSC Adv.* **2019**, *9*, 38531–38537, doi:10.1039/C9RA06385D.
41. Chen, Q.; Feng, N.B.; Huang, X.H.; Yao, Y.; Jin, Y.R.; Pan, W.; Liu, D. Humidity-Sensing Properties of a BiOCl-Coated Quartz Crystal Microbalance. *ACS Omega* **2020**, *5*, 18818–18825, doi:10.1021/acsomega.0c01946.
42. Rimeika, R.; Čiplys, D.; Poderys, V.; Rotomskis, R.; Shur, M.S. Fast-response surface acoustic wave humidity sensor based on hematoporphyrin film. *Sensors Actuators, B Chem.* **2009**, *137*, 592–596, doi:10.1016/j.snb.2009.02.009.
43. Su, Y.; Li, C.; Li, M.; Li, H.; Xu, S.; Qian, L.; Yang, B. Surface acoustic wave humidity sensor based on three-dimensional architecture graphene/PVA/SiO2 and its application for respiration monitoring. *Sensors Actuators, B Chem.* **2020**, *308*, 127693, doi:10.1016/j.snb.2020.127693.
44. Zhao, F.; Cheng, H.; Zhang, Z.; Jiang, L.; Qu, L. Direct Power Generation from a Graphene Oxide Film under Moisture. *Adv. Mater.* **2015**, *27*, 4351–4357, doi:10.1002/adma.201501867.
45. Shen, D.; Xiao, M.; Zou, G.; Liu, L.; Duley, W.W.; Zhou, Y.N. Self-Powered Wearable Electronics Based on Moisture Enabled Electricity Generation. *Adv. Mater.* **2018**, *30*, 1705925, doi:10.1002/adma.201705925.
46. Li, L.; Chen, Z.; Hao, M.; Wang, S.; Sun, F.; Zhao, Z.; Zhang, T. Moisture-Driven Power Generation for Multifunctional Flexible Sensing Systems. *Nano Lett.* **2019**, *19*, 5544–5552, doi:10.1021/acs.nanolett.9b02081.
47. Li, X.; Sun, Q.; Kan, Y.; Zhu, Y.; Pang, Z.; Li, M.; Jin, Y. Self-Powered Humidity Sensor Based on Polypyrrole/Melamine Aerogel for Real-Time Humidity Monitoring. *IEEE Sens. J.* **2021**, *21*, 2604–2609, doi:10.1109/JSEN.2020.3027743.
